# Supplementary material for: Nature-based interventions for individual, collective and planetary wellbeing: A protocol for a scoping review
Source: PLoS One. 2025 Apr 7;20(4):e0314591. doi: 10.1371/journal.pone.0314591 (PMC11975092; doi:10.1371/journal.pone.0314591)
Supplement: S1 Appendix — (DOCX) [file pone.0314591.s001.docx]

S1 Appendix. Search strategy.

A primary search string will be produced using all nature-based intervention search terms connected with the ‘OR’ operator. Secondary search strings will be generated by respectively combining all individual, collective, and planetary wellbeing search terms using the ‘OR’ operator. The primary search string will then be combined with each secondary search string in turn using the ‘AND’ operator. Database-specific search strategies may be seen in [S2 Table](#para10520_31285876182).
