# Supplementary material for: Nature-based interventions for individual, collective and planetary wellbeing: A protocol for a scoping review
Source: PLoS One. 2025 Apr 7;20(4):e0314591. doi: 10.1371/journal.pone.0314591 (PMC11975092; doi:10.1371/journal.pone.0314591)
Supplement: S2 Table — (DOCX) [file pone.0314591.s002.docx]

**S2 Table. Database-specific search strategies.**

| Database | Wellbeing domain | Search terms |
| --- | --- | --- |
| Scopus | Individual | ( TITLE-ABS-KEY ( "nature based therap*" OR "nature-based therap*" OR "nature based intervention*" OR "nature-based intervention*" OR “nature based activit*” OR “nature-based activit*” OR “nature therap*" OR “nature exposure” OR "green therap*" OR "green space*" OR "blue therap*" OR "blue space*" OR "brown therap*" OR "brown space*" OR "ecotherap*" OR "eco-therap*" OR "outdoor therap*" OR "outdoor healthcare" OR "outdoor behav* therap*" OR "environmental therap*" OR "green prescri*" OR “green gym*” OR “nature walk*” OR “simulated natural environment*” OR “animal assisted therap*” OR “animal-assisted therap*” OR “animal assisted intervention*” OR “animal-assisted intervention*” OR “animal assisted activit*” OR “animal-assisted activit*” OR “dog therap*” OR “canine therap*” OR “horse therap*” OR “equine therap*” OR “emotional support animal*” OR “emotional support pet” OR "forest therap*" OR "forest bathing" OR "forest-bathing" OR "shinrin-yoku" OR "shinrin yoku" OR "shinrinyoku" OR "horticultur* therap*" OR "horticultur* intervention*" OR "garden therap*" OR "garden intervention*" OR "wellbeing garden*" OR "well being garden*" OR "well-being garden*" OR "rehabilitation garden*" OR "care farm*" OR "community farm*" OR "wilderness-based therap*" OR "wilderness based therap*" OR "wilderness-adventure therap*" OR “wilderness therap*” OR "adventure therap*" OR "adventure-based therap*" OR "adventure based therap*" OR "adventure education" OR "therapeutic adventure") AND TITLE-ABS-KEY ( wellbeing OR "well being" OR "well-being" OR acceptance OR autonomy OR "self-determination" OR hope OR optimism OR humor OR humour OR spirituality OR "self-efficacy" OR "self-esteem" OR "self-identity" OR resilience OR intimacy OR "psychological health" OR "mental health" OR depression OR "anxiet*" OR "stress" OR "mental illness" OR psychopathology OR "life satisfaction" OR "quality of life" OR "QoL" OR "positive affect" OR "negative affect" OR "physical health" OR "heart rate variability" OR hrv OR cortisol OR "positive emotion*" OR "negative emotion*" OR "hedon*" OR "eudemon*" OR "eudaemon*" OR "eudaemon" OR happiness OR "disabilit*" OR functioning OR "engag*" OR "positive relationship*" OR meaning OR "accomplishment*" OR "achievement*" OR "biophil*" OR "bio phil*" OR "biophob*" OR "bio phob*" OR "restor*" OR "sense of purpose" OR benevolence OR "human value*" OR "personal value*" ) ) AND ( LIMIT-TO ( DOCTYPE , "ar" ) ) AND ( LIMIT-TO ( LANGUAGE , "English" ) ) AND ( LIMIT-TO ( SRCTYPE , "j" ) ) |
|  | Collective | ( TITLE-ABS-KEY ( "nature based therap*" OR "nature-based therap*" OR "nature based intervention*" OR "nature-based intervention*" OR "nature therap*" OR "green therap*" OR "green space*" OR "blue therap*" OR "blue space*" OR "brown therap*" OR "brown space*" OR "ecotherap*" OR "eco-therap*" OR "outdoor therap*" OR "outdoor healthcare" OR "outdoor behav* therap*" OR "environmental therap*" OR "green prescri*" OR "forest therap*" OR "forest bathing" OR "forest-bathing" OR "shinrin-yoku" OR "shinrin yoku" OR "shinrinyoku" OR "horticultur* therap*" OR "horticultur* intervention*" OR "garden therap*" OR "garden intervention*" OR "wellbeing garden*" OR "well being garden*" OR "well-being garden*" OR "rehabilitation garden*" OR "care farm*" OR "community farm*" OR "wilderness-based therap*" OR "wilderness based therap*" OR "wilderness-adventure therap*" OR “wilderness therap*” OR "adventure therap*" OR "adventure-based therap*" OR "adventure based therap*" OR "adventure education" OR "therapeutic adventure" ) AND TITLE-ABS-KEY ( "social wellbeing" OR "social well being" OR "social well-being" OR "social capital" OR "social participation" OR psychosocial OR "psycho-social" OR collective OR loneliness OR affiliation OR connection OR connectedness OR connectivity OR cohesion OR belonging ) ) AND ( LIMIT-TO ( DOCTYPE , "ar" ) ) AND ( LIMIT-TO ( LANGUAGE , "English" ) ) AND ( LIMIT-TO ( SRCTYPE , "j" ) ) |
|  | Planetary | ( TITLE-ABS-KEY ( "nature based therap*" OR "nature-based therap*" OR "nature based intervention*" OR "nature-based intervention*" OR "nature therap*" OR "green therap*" OR "green space*" OR "blue therap*" OR "blue space*" OR "brown therap*" OR "brown space*" OR "ecotherap*" OR "eco-therap*" OR "outdoor therap*" OR "outdoor healthcare" OR "outdoor behav* therap*" OR "environmental therap*" OR "green prescri*" OR "forest therap*" OR "forest bathing" OR "forest-bathing" OR "shinrin-yoku" OR "shinrin yoku" OR "shinrinyoku" OR "horticultur* therap*" OR "horticultur* intervention*" OR "garden therap*" OR "garden intervention*" OR "wellbeing garden*" OR "well being garden*" OR "well-being garden*" OR "rehabilitation garden*" OR "care farm*" OR "community farm*" OR "wilderness-based therap*" OR "wilderness based therap*" OR "wilderness-adventure therap*" OR “wilderness therap*” OR "adventure therap*" OR "adventure-based therap*" OR "adventure based therap*" OR "adventure education" OR "therapeutic adventure" ) AND TITLE-ABS-KEY ( "environmental attitude" OR "ecological attitude*" OR "ecological behav*" OR "environmental behav*" OR "nature conn*" OR "nature relat*" OR "nature ambivalence" OR "nature behav*" OR "proenvironmental" OR "pro-environmental" OR "pro environmental" OR "environmental steward*" OR "stewardship behav*" OR "environmental value*" OR "ecological value*" OR "biospheric value" OR "self-transcendent value*" OR "materialistic value*" OR universalism ) ) AND ( LIMIT-TO ( DOCTYPE , "ar" ) ) AND ( LIMIT-TO ( LANGUAGE , "English" ) ) AND ( LIMIT-TO ( SRCTYPE , "j" ) ) |
| EBSCO*host*:  APA | Individual | S1 = TI ( “nature based therap*” OR “nature-based therap*” OR “nature based intervention*” OR “nature-based intervention*” OR “nature based activit*” OR “nature-based activit*” OR “nature therap*” OR “nature exposure” OR “green therap*” OR “green space*” OR “blue therap*” OR “blue space*” OR “brown therap*” OR “brown space*” OR “ecotherap*” OR “eco-therap*” OR “outdoor therap*” OR “outdoor healthcare” OR “outdoor behav* therap*” OR “environmental therap*” OR “green prescri*” OR “green gym*” OR “nature walk*” OR “simulated natural environment*” OR “animal assisted therap*” OR “animal-assisted therap*” OR “animal assisted intervention*” “animal-assisted intervention*” OR “animal assisted activit*” OR “animal-assisted activit*” OR “dog therap*” OR “canine therap*” OR “horse therap*” OR “equine therap*” OR “emotional support animal*” OR “emotional support pet” OR “forest therap*” OR “forest bathing” OR “forest-bathing” OR “shinrin-yoku” OR “shinrin yoku” OR “shinrinyoku” OR “horticultur* therap*” OR “horticultur* intervention*” OR “garden therap*” OR “garden intervention*” OR “wellbeing garden*” OR “well being garden*” OR “well-being garden*” OR “rehabilitation garden*” OR “care farm*” OR “community farm*” OR “wilderness-based therap*” OR “wilderness based therap*” OR “wilderness-adventure therap*” OR “wilderness therap*” OR “adventure therap*” OR “adventure-based therap*” OR “adventure based therap*” OR “adventure education” OR “therapeutic adventure” ) OR AB ( “nature based therap*” OR “nature-based therap*” OR “nature based intervention*” OR “nature-based intervention*” OR “nature therap*” OR “green therap*” OR “green space*” OR “blue therap*” OR “blue space*” OR “brown therap*” OR “brown space*” OR “ecotherap*” OR “eco-therap*” OR “outdoor therap*” OR “outdoor healthcare” OR “outdoor behav* therap*” OR “environmental therap*” OR “green prescri*” OR “forest therap*” OR “forest bathing” OR “forest-bathing” OR “shinrin-yoku” OR “shinrin yoku” OR “shinrinyoku” OR “horticultur* therap*” OR “horticultur* intervention*” OR “garden therap*” OR “garden intervention*” OR “wellbeing garden*” OR “well being garden*” OR “well-being garden*” OR “rehabilitation garden*” OR “care farm*” OR “community farm*” OR “wilderness-based therap*” OR “wilderness based therap*” OR “wilderness-adventure therap*” OR “wilderness therap*” OR “adventure therap*” OR “adventure-based therap*” OR “adventure based therap*” OR “adventure education” OR “therapeutic adventure” )  S2 = DE “nature-based intervention”  S3 = S1 OR S2  S4 = TI ( wellbeing OR “well being” OR “well-being” OR acceptance OR autonomy OR “self-determination” OR hope OR optimism OR humor OR humour OR spirituality OR “self-efficacy” OR “self-esteem” OR “self-identity” OR resilience OR intimacy OR “psychological health” OR “mental health” OR depression OR “anxiet*” OR “stress” OR “mental illness” OR psychopathology OR “life satisfaction” OR “quality of life” OR “QoL” OR “positive affect” OR “negative affect” OR “physical health” OR “heart rate variability” OR HRV OR cortisol OR “positive emotion*” OR “negative emotion*” OR “hedon*” OR “eudemon*” OR “eudaemon*” OR “eudaemon” OR happiness OR “disabilit*” OR functioning OR “engag*” OR “positive relationship*” OR meaning OR “accomplishment*” OR “achievement*” OR “biophil*” OR “bio phil*” OR “biophob*” OR “bio phob*” OR “restor*” OR “sense of purpose” OR benevolence OR “human value*” OR “personal value*” ) OR AB ( wellbeing OR “well being” OR “well-being” OR acceptance OR autonomy OR “self-determination” OR hope OR optimism OR humor OR humour OR spirituality OR “self-efficacy” OR “self-esteem” OR “self-identity” OR resilience OR intimacy OR “psychological health” OR “mental health” OR depression OR “anxiet*” OR “stress” OR “mental illness” OR psychopathology OR “life satisfaction” OR “quality of life” OR “QoL” OR “positive affect” OR “negative affect” OR “physical health” OR “heart rate variability” OR HRV OR cortisol OR “positive emotion*” OR “negative emotion*” OR “hedon*” OR “eudemon*” OR “eudaemon*” OR “eudaemon” OR happiness OR “disabilit*” OR functioning OR “engag*” OR “positive relationship*” OR meaning OR “accomplishment*” OR “achievement*” OR “biophil*” OR “bio phil*” OR “biophob*” OR “bio phob*” OR “restor*” OR “sense of purpose” OR benevolence OR “human value*” OR “personal value*” )  S5 = DE “well being”  S6 = S4 OR S5  S7 = S3 AND S6  Search rules: Expanders: Apply equivalent subjects; Source Types: Peer Reviewed Journal, Dissertation Abstract; Language: [English](https://web.p.ebscohost.com/ehost/breadbox/clustersearch?cluster=ZL%20%22english%22&sid=5d68704e-111d-488d-aca7-019adcf715dc%40redis&vid=24); Search Mode: Proximity |
|  | Collective | S8 = TI ( “social wellbeing” OR “social well being” OR “social well-being” OR “social capital” OR “social participation” OR psychosocial OR “psycho-social” OR collective OR loneliness OR affiliation OR connection OR connectedness OR connectivity OR cohesion OR belonging ) OR AB ( “social wellbeing” OR “social well being” OR “social well-being” OR “social capital” OR “social participation” OR psychosocial OR “psycho-social” OR collective OR loneliness OR affiliation OR connection OR connectedness OR connectivity OR cohesion OR belonging )  S9 = DE “social connectedness”  S10 = S8 OR S9  S11 = S3 AND S10  Search rules: Expanders: Apply equivalent subjects; Source Types: Peer Reviewed Journal, Dissertation Abstract; Language: [English](https://web.p.ebscohost.com/ehost/breadbox/clustersearch?cluster=ZL%20%22english%22&sid=5d68704e-111d-488d-aca7-019adcf715dc%40redis&vid=24); Search Mode: Proximity |
|  | Planetary | S12 = TI ( “environmental attitude” OR “ecological attitude*” OR “ecological behav*” OR “environmental behav*” OR “nature conn*” OR “nature relat*” OR “nature ambivalence” OR “nature behav*” OR “proenvironmental” OR “pro-environmental” OR “pro environmental” OR “environmental steward*” OR “stewardship behav*” OR “environmental value*” OR “ecological value*” OR “biospheric value” OR “self-transcendent value*” OR “materialistic value*” OR universalism ) OR AB ( “environmental attitude” OR “ecological attitude*” OR “ecological behav*” OR “environmental behav*” OR “nature conn*” OR “nature relat*” OR “nature ambivalence” OR “nature behav*” OR “proenvironmental” OR “pro-environmental” OR “pro environmental” OR “environmental steward*” OR “stewardship behav*” OR “environmental value*” OR “ecological value*” OR “biospheric value” OR “self-transcendent value*” OR “materialistic value*” OR universalism  S13 = DE “Environmental Attitudes”  S14 = S12 OR S13  S15 = S3 AND S14  Search rules: Expanders: Apply equivalent subjects; Source Types: Peer Reviewed Journal, Dissertation Abstract; Language: [English](https://web.p.ebscohost.com/ehost/breadbox/clustersearch?cluster=ZL%20%22english%22&sid=5d68704e-111d-488d-aca7-019adcf715dc%40redis&vid=24); Search Mode: Proximity |
| MEDLINE | Individual | S1 = TI ( “nature based therap*” OR “nature-based therap*” OR “nature based intervention*” OR “nature-based intervention*” OR “nature based activit*” OR “nature-based activit*” OR “nature therap*” OR “nature exposure” OR “green therap*” OR “green space*” OR “blue therap*” OR “blue space*” OR “brown therap*” OR “brown space*” OR “ecotherap*” OR “eco-therap*” OR “outdoor therap*” OR “outdoor healthcare” OR “outdoor behav* therap*” OR “environmental therap*” OR “green prescri*” OR “green gym*” OR “nature walk*” OR “simulated natural environment*” OR “animal assisted therap*” OR “animal-assisted therap*” OR “animal assisted intervention*” “animal-assisted intervention*” OR “animal assisted activit*” OR “animal-assisted activit*” OR “dog therap*” OR “canine therap*” OR “horse therap*” OR “equine therap*” OR “emotional support animal*” OR “emotional support pet” OR “forest therap*” OR “forest bathing” OR “forest-bathing” OR “shinrin-yoku” OR “shinrin yoku” OR “shinrinyoku” OR “horticultur* therap*” OR “horticultur* intervention*” OR “garden therap*” OR “garden intervention*” OR “wellbeing garden*” OR “well being garden*” OR “well-being garden*” OR “rehabilitation garden*” OR “care farm*” OR “community farm*” OR “wilderness-based therap*” OR “wilderness based therap*” OR “wilderness-adventure therap*” OR “wilderness therap*” OR “adventure therap*” OR “adventure-based therap*” OR “adventure based therap*” OR “adventure education” OR “therapeutic adventure” ) OR AB ( “nature based therap*” OR “nature-based therap*” OR “nature based intervention*” OR “nature-based intervention*” OR “nature therap*” OR “green therap*” OR “green space*” OR “blue therap*” OR “blue space*” OR “brown therap*” OR “brown space*” OR “ecotherap*” OR “eco-therap*” OR “outdoor therap*” OR “outdoor healthcare” OR “outdoor behav* therap*” OR “environmental therap*” OR “green prescri*” OR “forest therap*” OR “forest bathing” OR “forest-bathing” OR “shinrin-yoku” OR “shinrin yoku” OR “shinrinyoku” OR “horticultur* therap*” OR “horticultur* intervention*” OR “garden therap*” OR “garden intervention*” OR “wellbeing garden*” OR “well being garden*” OR “well-being garden*” OR “rehabilitation garden*” OR “care farm*” OR “community farm*” OR “wilderness-based therap*” OR “wilderness based therap*” OR “wilderness-adventure therap*” OR “wilderness therap*” OR “adventure therap*” OR “adventure-based therap*” OR “adventure based therap*” OR “adventure education” OR “therapeutic adventure” )  S2 = TI ( wellbeing OR “well being” OR “well-being” OR acceptance OR autonomy OR “self-determination” OR hope OR optimism OR humor OR humour OR spirituality OR “self-efficacy” OR “self-esteem” OR “self-identity” OR resilience OR intimacy OR “psychological health” OR “mental health” OR depression OR “anxiet*” OR “stress” OR “mental illness” OR psychopathology OR “life satisfaction” OR “quality of life” OR “QoL” OR “positive affect” OR “negative affect” OR “physical health” OR “heart rate variability” OR HRV OR cortisol OR “positive emotion*” OR “negative emotion*” OR “hedon*” OR “eudemon*” OR “eudaemon*” OR “eudaemon” OR happiness OR “disabilit*” OR functioning OR “engag*” OR “positive relationship*” OR meaning OR “accomplishment*” OR “achievement*” OR “biophil*” OR “bio phil*” OR “biophob*” OR “bio phob*” OR “restor*” OR “sense of purpose” OR benevolence OR “human value*” OR “personal value*” ) OR AB ( wellbeing OR “well being” OR “well-being” OR acceptance OR autonomy OR “self-determination” OR hope OR optimism OR humor OR humour OR spirituality OR “self-efficacy” OR “self-esteem” OR “self-identity” OR resilience OR intimacy OR “psychological health” OR “mental health” OR depression OR “anxiet*” OR “stress” OR “mental illness” OR psychopathology OR “life satisfaction” OR “quality of life” OR “QoL” OR “positive affect” OR “negative affect” OR “physical health” OR “heart rate variability” OR HRV OR cortisol OR “positive emotion*” OR “negative emotion*” OR “hedon*” OR “eudemon*” OR “eudaemon*” OR “eudaemon” OR happiness OR “disabilit*” OR functioning OR “engag*” OR “positive relationship*” OR meaning OR “accomplishment*” OR “achievement*” OR “biophil*” OR “bio phil*” OR “biophob*” OR “bio phob*” OR “restor*” OR “sense of purpose” OR benevolence OR “human value*” OR “personal value*” )  S3 = S1 AND S2  Search rules: Limiters: Linked Full Text; Peer Reviewed; English Language; Expanders: Apply equivalent subjects; Search modes: Proximity |
|  | Collective | S4 = TI ( “social wellbeing” OR “social well being” OR “social well-being” OR “social capital” OR “social participation” OR psychosocial OR “psycho-social” OR collective OR loneliness OR affiliation OR connection OR connectedness OR connectivity OR cohesion OR belonging ) OR AB ( “social wellbeing” OR “social well being” OR “social well-being” OR “social capital” OR “social participation” OR psychosocial OR “psycho-social” OR collective OR loneliness OR affiliation OR connection OR connectedness OR connectivity OR cohesion OR belonging )  S5 = S1 AND S4  Search rules: Limiters: Linked Full Text; Peer Reviewed; English Language; Expanders: Apply equivalent subjects; Search modes: Proximity |
|  | Planetary | S6 = TI ( “environmental attitude” OR “ecological attitude*” OR “ecological behav*” OR “environmental behav*” OR “nature conn*” OR “nature relat*” OR “nature ambivalence” OR “nature behav*” OR “proenvironmental” OR “pro-environmental” OR “pro environmental” OR “environmental steward*” OR “stewardship behav*” OR “environmental value*” OR “ecological value*” OR “biospheric value” OR “self-transcendent value*” OR “materialistic value*” OR universalism ) OR AB ( “environmental attitude” OR “ecological attitude*” OR “ecological behav*” OR “environmental behav*” OR “nature conn*” OR “nature relat*” OR “nature ambivalence” OR “nature behav*” OR “proenvironmental” OR “pro-environmental” OR “pro environmental” OR “environmental steward*” OR “stewardship behav*” OR “environmental value*” OR “ecological value*” OR “biospheric value” OR “self-transcendent value*” OR “materialistic value*” OR universalism  S7 = S1 AND S6  Search rules: Limiters: Linked Full Text; Peer Reviewed; English Language; Expanders: Apply equivalent subjects; Search modes: Proximity |
| Web of Science: Core Collection | Individual | S1 = TS = (“nature based therap*” OR “nature-based therap*” OR “nature based intervention*” OR “nature-based intervention*” OR “nature based activit*” OR “nature-based activit*” OR “nature therap*” OR “nature exposure” OR “green therap*” OR “green space*” OR “blue therap*” OR “blue space*” OR “brown therap*” OR “brown space*” OR “ecotherap*” OR “eco-therap*” OR “outdoor therap*” OR “outdoor healthcare” OR “outdoor behav* therap*” OR “environmental therap*” OR “green prescri*” OR “green gym*” OR “nature walk*” OR “simulated natural environment*” OR “animal assisted therap*” OR “animal-assisted therap*” OR “animal assisted intervention*” “animal-assisted intervention*” OR “animal assisted activit*” OR “animal-assisted activit*” OR “dog therap*” OR “canine therap*” OR “horse therap*” OR “equine therap*” OR “emotional support animal*” OR “emotional support pet” OR “forest therap*” OR “forest bathing” OR “forest-bathing” OR “shinrin-yoku” OR “shinrin yoku” OR “shinrinyoku” OR “horticultur* therap*” OR “horticultur* intervention*” OR “garden therap*” OR “garden intervention*” OR “wellbeing garden*” OR “well being garden*” OR “well-being garden*” OR “rehabilitation garden*” OR “care farm*” OR “community farm*” OR “wilderness-based therap*” OR “wilderness based therap*” OR “wilderness-adventure therap*” OR “wilderness therap*” OR “adventure therap*” OR “adventure-based therap*” OR “adventure based therap*” OR “adventure education” OR “therapeutic adventure”) AND Article (Document Types) and English (Languages)  S2 = TS=(wellbeing OR “well being” OR “well-being” OR acceptance OR autonomy OR “self-determination” OR hope OR optimism OR humor OR humour OR  spirituality OR “self-efficacy” OR  “self-esteem” OR “self-identity” OR resilience OR intimacy OR “psychological health” OR “mental health” OR depression OR “anxiet*” OR “stress” OR “mental illness” OR psychopathology OR “life satisfaction” OR “quality of life” OR “QoL” OR “positive affect” OR “negative affect” OR “physical health” OR “heart rate variability” OR HRV OR cortisol OR “positive emotion*” OR “negative emotion*” OR “hedon*” OR “eudemon*” OR “eudaemon*” OR “eudaemon” OR happiness OR “disabilit*” OR functioning OR “engag*” OR “positive relationship*” OR meaning OR “accomplishment*” OR “achievement*” OR “biophil*” OR “bio phil*” OR “biophob*” OR “bio phob*” OR “restor*” OR “sense of purpose” OR benevolence OR “human value*” OR “personal value*”) AND Article (Document Types) and English (Languages)  S3 = S1 AND S2 |
|  | Collective | S4 = TS=(“social wellbeing” OR “social well being” OR “social well-being” OR “social capital” OR “social participation” OR psychosocial OR “psycho-social” OR collective OR loneliness OR affiliation OR connection OR connectedness OR connectivity OR cohesion OR belonging) AND Article (Document Types) and English (Languages)  S5 = S4 AND S1 |
|  | Planetary | S6 = TS= (“environmental attitude” OR “ecological attitude*” OR “ecological behav*” OR “environmental behav*” OR “nature conn*” OR “nature relat*” OR “nature ambivalence” OR “nature behav*” OR “proenvironmental” OR “pro-environmental” OR “pro environmental” OR “environmental steward*” OR “stewardship behav*” OR “environmental value*” OR “ecological value*” OR “biospheric value” OR “self-transcendent value*” OR “materialistic value*” OR universalism) AND Article (Document Types) and English (Languages)  S7 = S1 AND S6 |
| ProQuest Dissertations & Theses Global | Individual | noft("nature based therap*" OR "nature-based therap*" OR "nature based intervention*" OR "nature-based intervention*" OR “nature based activit*” OR “nature-based activit*” OR “nature therap*” OR “nature exposure” OR "green therap*" OR "green space*" OR "blue therap*" OR "blue space*" OR "brown therap*" OR "brown space*" OR "ecotherap*" OR "eco-therap*" OR "outdoor therap*" OR "outdoor healthcare" OR "outdoor behav* therap*" OR "environmental therap*" OR "green prescri*" OR “green gym*” OR “nature walk*” OR “simulated natural environment*” OR “animal assisted therap*” OR “animal-assisted therap*” OR “animal assisted intervention*” OR “animal-assisted intervention*” OR “animal assisted activit*” OR “animal-assisted activit*” OR “dog therap*” OR “canine therap*” OR “horse therap*” OR “equine therap*” OR “emotional support animal*” OR “emotional support pet” OR "forest therap*" OR "forest bathing" OR "forest-bathing" OR "shinrin-yoku" OR "shinrin yoku" OR "shinrinyoku" OR "horticultur* therap*" OR "horticultur* intervention*" OR "garden therap*" OR "garden intervention*" OR "wellbeing garden*" OR "well being garden*" OR "well-being garden*" OR "rehabilitation garden*" OR "care farm*" OR "community farm*" OR "wilderness-based therap*" OR "wilderness based therap*" OR "wilderness-adventure therap*" OR “wilderness therap*” OR "adventure therap*" OR "adventure-based therap*" OR "adventure based therap*" OR "adventure education" OR "therapeutic adventure"  ) AND noft(wellbeing OR "well being" OR "well-being" OR acceptance OR autonomy OR "self-determination" OR hope OR optimism OR humor OR humour OR spirituality OR "self-efficacy" OR "self-esteem" OR "self-identity" OR resilience OR intimacy OR "psychological health" OR "mental health" OR depression OR "anxiet*" OR "stress" OR "mental illness" OR psychopathology OR "life satisfaction" OR "quality of life" OR "QoL" OR "positive affect" OR "negative affect" OR "physical health" OR "heart rate variability" OR hrv OR cortisol OR "positive emotion*" OR "negative emotion*" OR "hedon*" OR "eudemon*" OR "eudaemon*" OR "eudaemon" OR happiness OR "disabilit*" OR functioning OR "engag*" OR "positive relationship*" OR meaning OR "accomplishment*" OR "achievement*" OR "biophil*" OR "bio phil*" OR "biophob*" OR "bio phob*" OR "restor*" OR "sense of purpose" OR benevolence OR "human value*" OR "personal value*")  Search rules: Full text; Language: [English](https://web.p.ebscohost.com/ehost/breadbox/clustersearch?cluster=ZL%20%22english%22&sid=5d68704e-111d-488d-aca7-019adcf715dc%40redis&vid=24) |
|  | Collective | noft("nature based therap*" OR "nature-based therap*" OR "nature based intervention*" OR "nature-based intervention*" OR “nature based activit*” OR “nature-based activit*” OR “nature therap*” OR “nature exposure” OR "green therap*" OR "green space*" OR "blue therap*" OR "blue space*" OR "brown therap*" OR "brown space*" OR "ecotherap*" OR "eco-therap*" OR "outdoor therap*" OR "outdoor healthcare" OR "outdoor behav* therap*" OR "environmental therap*" OR "green prescri*" OR “green gym*” OR “nature walk*” OR “simulated natural environment*” OR “animal assisted therap*” OR “animal-assisted therap*” OR “animal assisted intervention*” OR “animal-assisted intervention*” OR “animal assisted activit*” OR “animal-assisted activit*” OR “dog therap*” OR “canine therap*” OR “horse therap*” OR “equine therap*” OR “emotional support animal*” OR “emotional support pet” OR "forest therap*" OR "forest bathing" OR "forest-bathing" OR "shinrin-yoku" OR "shinrin yoku" OR "shinrinyoku" OR "horticultur* therap*" OR "horticultur* intervention*" OR "garden therap*" OR "garden intervention*" OR "wellbeing garden*" OR "well being garden*" OR "well-being garden*" OR "rehabilitation garden*" OR "care farm*" OR "community farm*" OR "wilderness-based therap*" OR "wilderness based therap*" OR "wilderness-adventure therap*" OR “wilderness therap*” OR "adventure therap*" OR "adventure-based therap*" OR "adventure based therap*" OR "adventure education" OR "therapeutic adventure") AND noft("social wellbeing" OR "social well being" OR "social well-being" OR "social capital" OR "social participation" OR psychosocial OR "psycho-social" OR collective OR loneliness OR affiliation OR connection OR connectedness OR connectivity OR cohesion OR belonging )  Search rules: Full text; Language: [English](https://web.p.ebscohost.com/ehost/breadbox/clustersearch?cluster=ZL%20%22english%22&sid=5d68704e-111d-488d-aca7-019adcf715dc%40redis&vid=24) |
|  | Planetary | noft("nature based therap*" OR "nature-based therap*" OR "nature based intervention*" OR "nature-based intervention*" OR “nature based activit*” OR “nature-based activit*” OR “nature therap*” OR “nature exposure” OR "green therap*" OR "green space*" OR "blue therap*" OR "blue space*" OR "brown therap*" OR "brown space*" OR "ecotherap*" OR "eco-therap*" OR "outdoor therap*" OR "outdoor healthcare" OR "outdoor behav* therap*" OR "environmental therap*" OR "green prescri*" OR “green gym*” OR “nature walk*” OR “simulated natural environment*” OR “animal assisted therap*” OR “animal-assisted therap*” OR “animal assisted intervention*” OR “animal-assisted intervention*” OR “animal assisted activit*” OR “animal-assisted activit*” OR “dog therap*” OR “canine therap*” OR “horse therap*” OR “equine therap*” OR “emotional support animal*” OR “emotional support pet” OR "forest therap*" OR "forest bathing" OR "forest-bathing" OR "shinrin-yoku" OR "shinrin yoku" OR "shinrinyoku" OR "horticultur* therap*" OR "horticultur* intervention*" OR "garden therap*" OR "garden intervention*" OR "wellbeing garden*" OR "well being garden*" OR "well-being garden*" OR "rehabilitation garden*" OR "care farm*" OR "community farm*" OR "wilderness-based therap*" OR "wilderness based therap*" OR "wilderness-adventure therap*" OR “wilderness therap*” OR "adventure therap*" OR "adventure-based therap*" OR "adventure based therap*" OR "adventure education" OR "therapeutic adventure") AND noft((“environmental attitude” OR “ecological attitude*” OR “ecological behav*” OR “environmental behav*” OR “nature conn*” OR “nature relat*” OR “nature ambivalence” OR “nature behav*” OR “proenvironmental” OR “pro-environmental” OR “pro environmental” OR “environmental steward*” OR “stewardship behav*” OR “environmental value*” OR “ecological value*” OR “biospheric value” OR “self-transcendent value*” OR “materialistic value*” OR universalism) )  Search rules: Full text; Language: [English](https://web.p.ebscohost.com/ehost/breadbox/clustersearch?cluster=ZL%20%22english%22&sid=5d68704e-111d-488d-aca7-019adcf715dc%40redis&vid=24) |
